# Effect of add-on naldemedine treatment in patients with cancer and opioid-induced constipation insufficiently responding to magnesium oxide: a pooled, subgroup analysis of two randomized controlled trials

**DOI:** 10.1093/jjco/hyae135

**Published:** 2024-10-01

**Authors:** Takaomi Kessoku, Toshikazu Akamatsu, Yasuhide Morioka, Takaaki Yokota, Masayuki Kobayashi, Kohei Uchida, Yuichi Koretaka, Atsushi Nakajima

**Affiliations:** Department of Palliative Medicine and Gastroenterology, International University of Health and Welfare Narita Hospital, Narita, Chiba, Japan; Department of Gastroenterology, International University of Health and Welfare Graduate School of Medicine, Narita, Chiba, Japan; Department of Gastroenterology and Hepatology, Yokohama City University Graduate School of Medicine, Yokohama, Japan; Medical Affairs Department, Shionogi & Co., Ltd, Chuo-ku, Osaka, Japan; Medical Affairs Department, Shionogi & Co., Ltd, Chuo-ku, Osaka, Japan; Clinical Research Department, Shionogi & Co., Ltd, Chuo-ku, Osaka, Japan; Data Science Department, Shionogi & Co., Ltd, Chuo-ku, Osaka, Japan; Business System Department, INFRONT Inc., Osaka, Japan; Data Science Department, Shionogi & Co., Ltd, Chuo-ku, Osaka, Japan; Department of Gastroenterology and Hepatology, Yokohama City University Graduate School of Medicine, Yokohama, Japan

**Keywords:** cancer management, clinical management, clinical trials, drug discovery and delivery, quality of life

## Abstract

**Objective:**

To evaluate the additive effect of naldemedine tosylate (naldemedine) on opioid-induced constipation in cancer patients insufficiently responding to magnesium oxide treatment.

**Methods:**

We combined two randomized, double-blind, placebo-controlled, phase IIb and III trials of naldemedine and conducted a *post hoc* subgroup analysis. We evaluated the effect and safety of naldemedine in 116 patients who received naldemedine in addition to magnesium oxide (naldemedine group) and 117 patients who received placebo in addition to magnesium oxide (placebo group). Both groups included patients insufficiently responding to magnesium oxide for opioid-induced constipation. Effect was assessed using spontaneous bowel movement responder rate, complete spontaneous bowel movement responder rate, changes in spontaneous bowel movements and complete spontaneous bowel movements. Safety was also assessed.

**Results:**

During the 2-week treatment period, the responder rates for spontaneous bowel movement and complete spontaneous bowel movement were 73.3 and 43.1% in naldemedine group, respectively, which were significantly higher (*P* < 0.0001) than 41.9 and 14.5% in placebo group, respectively. Median time to first spontaneous bowel movement and first complete spontaneous bowel movement was significantly shorter (*P* < 0.0001) in the naldemedine group (4.0 and 21.3 h, respectively) than in the placebo group (27.7 and 211.7 h, respectively). The incidence of adverse events and diarrhoea was significantly higher (*P* < 0.05) in the naldemedine group than in the placebo group, while the incidence of serious adverse events and severe diarrhoea was not significantly different between the naldemedine and placebo groups.

**Conclusion:**

The study suggested the addition of naldemedine as an effective treatment option for opioid-induced constipation in cancer patients insufficiently responding to magnesium oxide treatment.

## Teaser

Naldemedine significantly improved the SBM/CSBM responder rates in cancer patients with opioid-induced constipation unresponsive to magnesium oxide, with shorter time to first SBM/CSBM, per pooled analysis of two randomized placebo-controlled trials.

## Introduction

Opioids have remained the drug of choice for managing moderate to severe cancer pain (1). However, patients taking opioids for pain management experience major side effects, such as sedation, nausea, vomiting and constipation, in addition to respiratory depression, muscular rigidity, dysphoria and miosis (2). Gastrointestinal (GI) side effects are collectively referred to as opioid-induced bowel dysfunction (OIBD), which is characterized by dry mouth, increased gastric reflux, bloating, abdominal distension, hard and dry stools and a sense of incomplete evacuation. The most common symptom of OIBD is opioid-induced constipation (OIC) mediated by μ-opioid receptors (MOR) in the enteric system (3).

Previous studies reported that chronic OIC negatively affected pain management and health-related quality of life (QOL) of patients, with potential increase in healthcare resource utilization (4–7). OIC affects 55–65% of patients with cancer and can reduce their QOL (8–11). Exercise, stimulant laxatives, high fibre intake and increased hydration are used prophylactically or as first-line treatments of OIC; however, they do not target the underlying mechanism of OIC and usually provide inadequate or inconsistent relief (4,12). Indeed, previous studies reported cumulative lower incidence (34–48%) of OIC in patients who received prophylactic laxative medications than in those who did not (55–65%) (8,9).

Magnesium oxide, an osmotic laxative, is the most commonly used first-line agent for the treatment of OIC in Japan, due to its high usage experience and low cost (13,14). Moreover, clinical guidelines from the Japanese Society of Palliative Medicine recommend the use of magnesium oxide as the first-line agent (15). However, limited evidence supports its use in OIC. Being an osmotic laxative that does not target MOR, it has limited efficacy, and often increases patients’ burden by causing unpleasant side effects and negatively impacting their QOL (16). Its limited efficacy in OIC may be further reduced with concomitant use of drugs (commonly prescribed in cancer patients) such as proton pump inhibitors and histamine H_2_ receptor antagonists (14).

Peripherally acting MOR antagonists (PAMORAs) block opioid actions at MOR in the GI tract, resulting in the reduction of OIC without having analgesic effects of opioids (17). As per recommendations of the subgroup of Multinational Association for Supportive Care in Cancer, PAMORAs should always be administered for the treatment of OIC in patients with cancer (18). Other recommendations suggest the use of PAMORAs as the second or later line drugs in cancer patients when other laxatives do not completely relieve OIC (19). Naldemedine, one of the PAMORAs, acts by inhibiting MOR in the GI tract and reduces OIC in patients with cancer and noncancer pain (20,21).

Previous clinical trials have demonstrated the efficacy and safety of naldemedine in cancer patients with OIC. A phase IIb dose-determining trial conducted on cancer patients with OIC showed that naldemedine treatment (0.2 mg dose) significantly improved (*P* < 0.001) the change in frequency of spontaneous bowel movements (SBMs), SBM responder rates and the change in frequency of complete SBMs (CSBMs) during the 2-week treatment period compared with placebo (22). Furthermore, a double-blind, phase III trial (COMPOSE-4) in cancer patients with OIC reported that the 2-week treatment significantly increased (*P* < 0.0001) the proportion of SBM responders in the naldemedine (0.2 mg) group compared with the placebo group (23). The open-label extension of this trial (COMPOSE-5; additional 12 weeks) has also confirmed the efficacy and safety of naldemedine for OIC in patients with cancer (23). In COMPOSE-4, changes from baseline to 2 weeks in naldemedine vs. placebo groups in the mean overall scores for Patient Assessment of Constipation Symptoms (PAC-SYM) or Patient Assessment of Constipation Quality of Life (PAC-QOL) were not significant. However, in COMPOSE-5, patients showed significant improvements (*P* < 0.0001) in overall mean scores for both PAC-SYM and PAC-QOL from baseline to all time points (24).

A pooled subgroup analysis of the above two randomized controlled trials showed that the efficacy and safety of naldemedine (0.2 mg) in patients with OIC and cancer were consistent across the subgroups considering relevant baseline characteristics (25). Proportions of SBM responders by prior use of laxatives (yes/no) and by the type of prior regular laxative use (magnesium oxide, sennoside A + B, or other laxative) were in general comparable. In addition, proportions of patients with diarrhoea, a major side effect, were similar in these subgroups (25). The above pooled analysis indicated that naldemedine treatment improved the SBM responses in patients with prior use of magnesium oxide. However, it is unclear whether naldemedine is efficacious irrespective of the ongoing concomitant use of magnesium oxide with naldemedine treatment. Therefore, it is essential to assess the effect of add-on naldemedine administered concomitantly with the ongoing magnesium oxide treatment in cancer patients with OIC insufficiently responding to magnesium oxide.

While the previous two trials focused on efficacy and safety of naldemedine for the treatment of OIC in patients with cancer, this study aimed to evaluate the additive effect of naldemedine for OIC in cancer patients insufficiently responding to magnesium oxide treatment. In this *post hoc* pooled analysis study, the effect and safety of naldemedine were compared with placebo in a subgroup of patients who were taking magnesium oxide before and during naldemedine treatment.

## Patients and methods

### Study design

This study is a *post hoc* pooled subgroup analysis using data from patients from two randomized, double-blind, placebo-controlled trials in Japan evaluating the effect and safety of three doses of naldemedine: a phase IIb trial (Clinical trial registration number: jRCT2080221471) (22) and a phase III trial COMPOSE-4 (Clinical trial registration number: jRCT2080222291) (23,24). The trials included adult patients with cancer (that was unlikely to directly affect the GI system) and OIC (experiencing ≤5 SBMs and straining, sense of incomplete evacuation and/or hard stools in ≥25% of all bowel movements in the 2 weeks prior to treatment enrollment). For inclusion in these trials, the patient had to have an Eastern Cooperative Oncology Group (ECOG) performance status ≤2 and have received opioids for ≥2 weeks prior to screening.

The phase IIb trial (22) included 225 cancer patients with OIC divided into four groups (naldemedine 0.1 mg, 55 patients; naldemedine 0.2 mg, 58 patients; naldemedine 0.4 mg, 56 patients; placebo, 56 patients). Treatment was administered orally once daily for 2 weeks and patients were followed up for 4 weeks. The primary end point was change in SBM frequency per week, while the secondary end points were SBM responder rate, change in SBM frequency without straining and change in CSBM frequency from baseline.

In the phase III trial COMPOSE-4 (193 patients; naldemedine 0.2 mg group, 97 patients; and placebo, 96 patients), the primary end point was a proportion of SBM responders during the 2-week treatment period (23). Patients in the COMPOSE-4 trial answered the questionnaires of PAC-SYM and PAC-QOL. Overall PAC-SYM score comprised three domains: abdominal, rectal and stool symptoms, while overall PAC-QOL score consisted of four domains: physical discomfort, psychosocial discomfort, satisfaction and worries and concerns (24).

### Patients

This subgroup analysis included patients who participated in the above phase IIb and III clinical trials in either naldemedine or placebo group and who had been using magnesium oxide before starting naldemedine and continued its use concomitantly with naldemedine treatment. A total of 418 patients were allocated into the following two groups: naldemedine group (266 patients; 169 from phase IIb and 97 from phase III study) and placebo group (152 patients; 56 from phase IIb and 96 from phase III study) (Table 1, Fig. S1). Of these, patients who had been taking magnesium oxide before and after starting naldemedine treatment (0.2 mg) were identified as the naldemedine group (116 patients; 53 from phase IIb and 63 from phase III study) and the placebo group (117 patients; 50 from phase IIb and 67 from phase III study) (Table 1, Fig. S1).

**Table 1:** Patient selection

**Patients**	**Phase IIb**		**Phase III**		**Pooled**		
	**Naldemedine group**	**Placebo group**	**Naldemedine group**	**Placebo group**	**Naldemedine group**	**Placebo group**	**Total**
Patients who received naldemedine or placebo (*n*)	169	56	97	96	266	152	418
Patients who received naldemedine (0.2 mg) or placebo with prior and concomitant regular use of magnesium oxide (*n*)	53	50	63	67	116	117	233

### Outcomes

Outcomes in this subgroup analysis were defined according to the phase IIb and III clinical trials. Briefly, effect was assessed using SBM responder rate, CSBM responder rate, changes of SBMs and CSBMs, SBMs with Bristol stool score (BSS) 3 or 4 and without straining, changes of PAC-SYM score, PAC-SYM responder rate, changes of PAC-QOL score, and PAC-QOL responder rate during each week of naldemedine treatment and the 2-week treatment period. SBM responders were defined as patients with ≥3 SBMs per week and an increase of ≥1 SBM per week from baseline, while CSBM responders were defined as patients with SBM accompanied by feelings of complete evacuation. Patients who had improved PAC-SYM overall score ≥1 from baseline were identified as PAC-SYM responders. Patients who had improved PAC-QOL dissatisfaction score ≥1 from baseline were identified as PAC-QOL responders. Safety was assessed using incidences of death, adverse events (AEs), serious AEs (SAEs), diarrhoea and severe diarrhoea.

### Statistical analysis

Data for quantitative variables were expressed as mean and standard deviation (SD) or for change from baseline as least square (LS) mean and standard error (SE). Mean differences between treatment groups and mean changes from baseline scores were compared using Welch *t*-test. Data for qualitative variables were expressed as proportion (%) and 95% confidence intervals (CI) of proportions were calculated using the method described by Koch et al. (26). Differences in proportions (e.g. responder rates for the naldemedine group vs. placebo) were evaluated using Cochran–Mantel–Haenszel test adjusted by study. Time to first SBM and CSBM was estimated based on Kaplan–Meier estimates of time to first event and reported as median (95% CI). *P* value in pooled studies was calculated by the generalized Wilcoxon test adjusted by study. The analysis of covariance models was used to assess the changes of frequencies of SBMs, CSBMs, SBMs with BSS of 3 or 4 and without straining, or the change in the number of days with at least 1 SBM per week and had the terms for treatment group as a fixed effect and baseline value as a covariate. In pooled studies, study was added as a covariate. AE incidences for the naldemedine group vs. placebo were compared using Fisher exact test. For all two-sided tests, statistical significance level was set at 0.05. Statistical analyses were carried out using SAS 9.4 (SAS Institute, Cary, NC).

**Table 2:** Patient demographics and baseline characteristics

**Characteristic**	**Naldemedine group** **(*n* = 116)**	**Placebo group** **(*n* = 117)**
*Age, years*		
Mean (SD)	63.6 (9.9)	64.8 (10.7)
*Age categories, n (%), years*		
<40	4 (3.4)	2 (1.7)
≥40 to <50	8 (6.9)	11 (9.4)
≥50 to <65	46 (39.7)	39 (33.3)
≥65 to <75	48 (41.4)	41 (35.0)
≥75	10 (8.6)	24 (20.5)
*Sex, n (%)*		
Male	70 (60.3)	74 (63.2)
Female	46 (39.7)	43 (36.8)
*Body weight (kg)*		
Mean (SD)	55.2 (9.3)	55.1 (11.0)
*Body mass index (kg/m^2^)*		
Mean (SD)	21.6 (3.4)	21.1 (3.8)
*Body mass index categories, n (%)*		
<18.5	20 (17.2)	28 (23.9)
≥18.5 to <25.0	78 (67.2)	73 (62.4)
≥25.0 to <30.0	16 (13.8)	13 (11.1)
≥30.0	2 (1.7)	3 (2.6)
*Race, n (%)*		
Asian	116 (100.0)	117 (100.0)
*Country, n (%)*		
Japan	113 (97.4)	116 (99.1)
Korea	3 (2.6)	1 (0.9)
*Regular opioid use per day at baseline* ^a^ *, mg (%)*		
Mean (SD)	71.1 (70.2)	80.7 (106.8)
*Regular opioid use per day at baseline categories, n (%)*		
<30	29 (25.0)	26 (22.2)
≥30 to <60	30 (25.9)	39 (33.3)
≥60 to <120	35 (30.2)	30 (25.6)
≥120	22 (19.0)	22 (18.8)
*Use of rescue laxative per week at baseline (times)*		
Mean (SD)	5.7 (4.3)	6.1 (4.9)
*Category of patient, n (%)*		
Inpatient	27 (23.3)	29 (24.8)
Outpatient	89 (76.7)	88 (75.2)
*Primary tumour diagnosed, n (%)*		
Lung	43 (37.1)	59 (50.4)
Breast	25 (21.6)	26 (22.2)
Large intestine	4 (3.4)	1 (0.9)
Other	44 (37.9)	31 (26.5)
*Presence of metastasis, n (%)*		
Yes	99 (85.3)	106 (90.6)
No	17 (14.7)	11 (9.4)
*Eastern Cooperative Oncology Group performance status, n (%)*		
0	25 (21.6)	39 (33.3)
1	69 (59.5)	62 (53.0)
2	22 (19.0)	16 (13.7)

SD, standard deviation.

^a^Dose of opioid analgesics was used by converting into equivalent oral morphine dose.

## Results

### Patient demographic and baseline characteristics

Patient demographic and baseline characteristics are presented in Table 2 and were in general similar across the two groups. Of 233 included patients, the mean ± SD age was 63.6 ± 9.9 and 64.8 ± 10.7 years for naldemedine (*n* = 116) and placebo (*n* = 117) groups; proportion of males was 60.3 and 63.2%, respectively. Patients aged ≥65 years were 50.0% in the naldemedine group and 55.5% in the placebo group. Only 3.4% patients in the naldemedine group and 1.7% in the placebo group were aged <40 years. Regular mean (SD) dose of opioid per day at baseline was 71.1 (70.2) mg in the naldemedine group vs. 80.7 (106.8) mg in the placebo group. The mean frequency of rescue laxative use per week at baseline was 5.7 times in the naldemedine group and 6.1 times in the placebo group. Of 116 patients in the naldemedine group, the majority of patients (81.1%) were categorized as ECOG performance status 0 or 1, while the proportion was 86.3% for the placebo group. Patient demographic and baseline characteristics of phase IIb and III studies are shown in Table S1. The regular and rescue laxatives used by patients in the naldemedine and the placebo groups are shown in Table S2.

### Proportion of SBM and CSBM responders and change in SBM and CSBM responder rate

**Figure 1:**
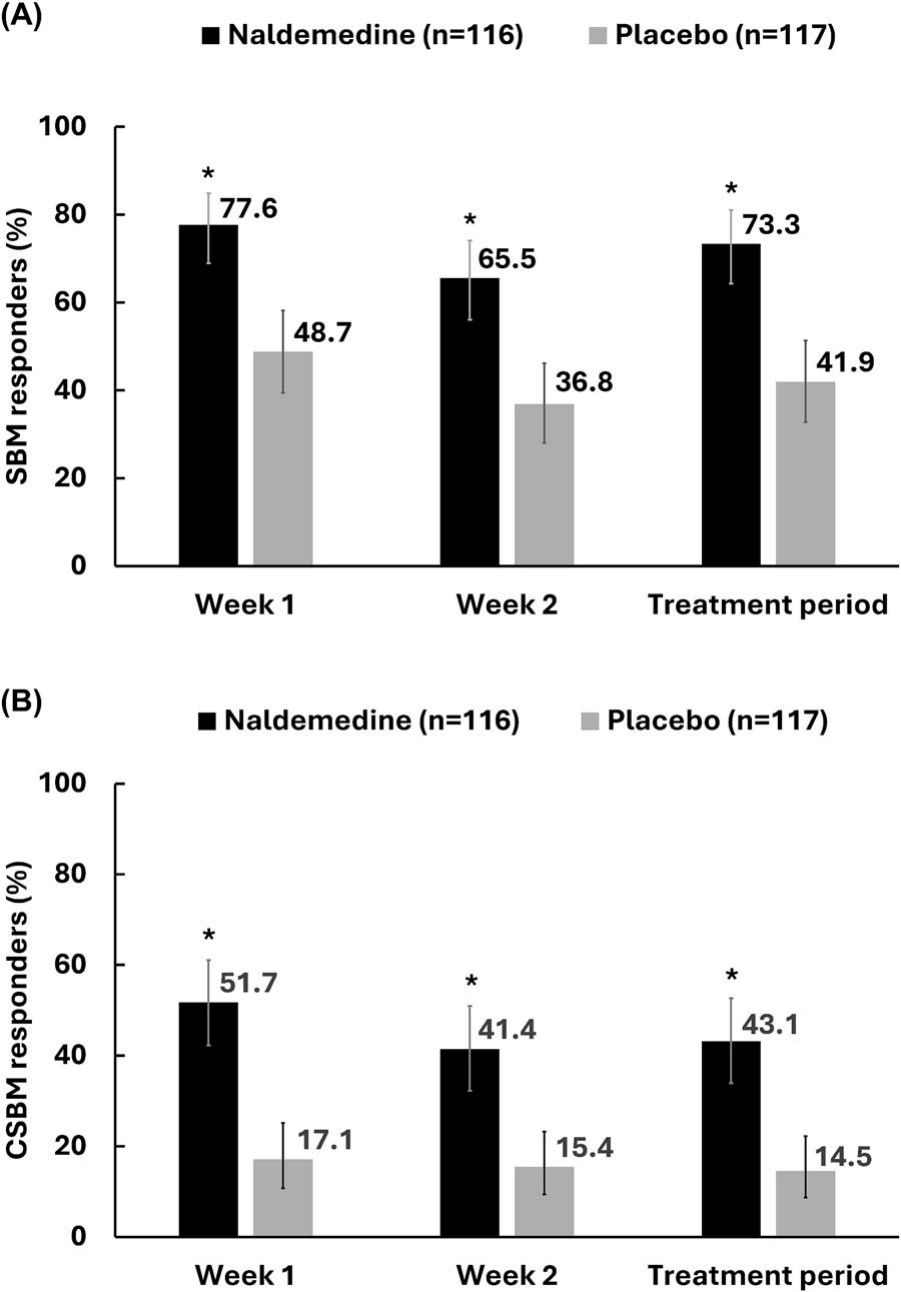
Proportion of SBM responders at week 1, week 2 and over 2-week treatment period (A); proportion of CSBM responders at week 1, week 2 and over 2-week treatment period (B); proportion ± 95% CI. ^*^P < 0.0001 vs. placebo; CI, confidence interval; CSBM, complete spontaneous bowel movement; SBM, spontaneous bowel movement.

Over a treatment period of 2 weeks, the proportion of SBM responders (95% CI) was 73.3% (64.26%, 81.07%) in the naldemedine group vs. 41.9% (32.82%, 51.36%) in the placebo group. The proportion of CSBM responders (95% CI) was 43.1% (33.94%, 52.62%) in the naldemedine group vs. 14.5% (8.70%, 22.24%) in the placebo group (Fig. 1). Significant differences were observed (*P* < 0.0001) in SBM responders and CSBM responders in the naldemedine group compared with the placebo group at 1-week, 2-week and over the 2-week treatment periods.

### Time to first SBM and CSBM

**Figure 2:**
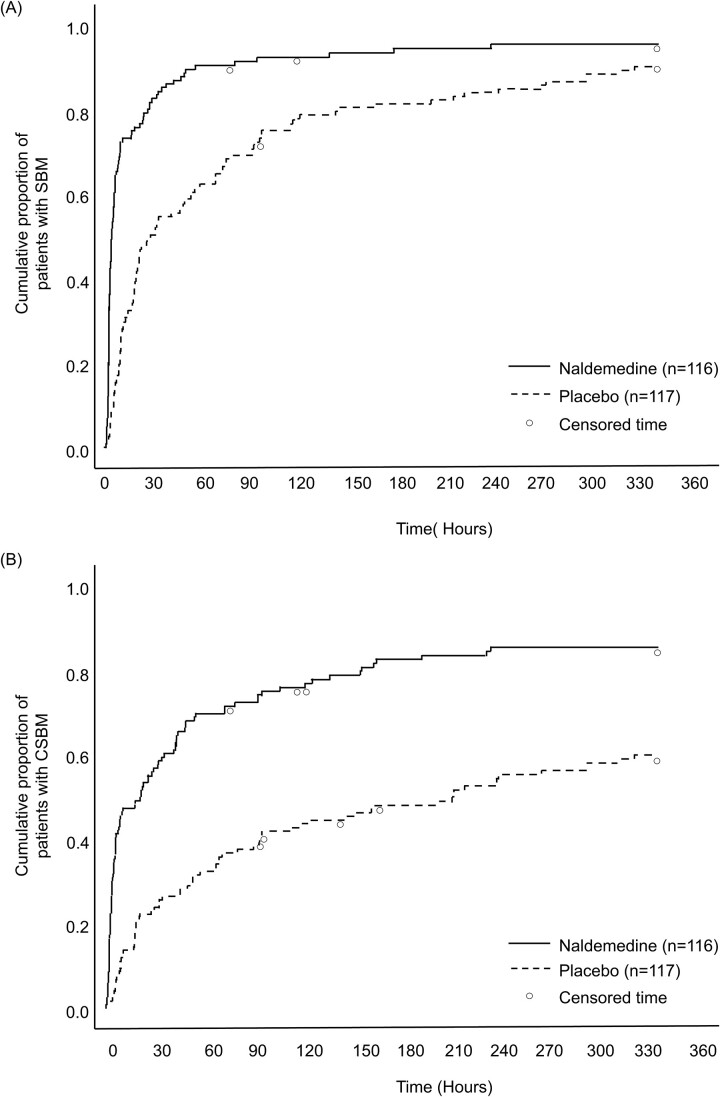
Kaplan–Meier curve of time to first SBM (A) or CSBM (B). Circles represent censored time. The time to the first SBM or CSBM was censored for subjects who withdrew from the study before an SBM or CSBM was observed, or if no SBM or CSBM occurred during the 2-week treatment period. CSBM, complete spontaneous bowel movement; SBM, spontaneous bowel movement.

The time to first SBM and first CSBM were significantly shorter (*P* < 0.0001) in the naldemedine group than in the placebo group. Median time (95% CI) to first SBM and first CSBM were 4.0 h (3.0h, 5.3 h) and 21.3 h (6.3 h, 34.4 h) in the naldemedine group and 27.7 h (18.7h, 50.0 h) and 211.7 h (94.1h, 311.5 h) in the placebo group, respectively (Fig. 2).

### Proportion of subjects with ≥1 SBM or ≥1 CSBM

**Figure 3:**
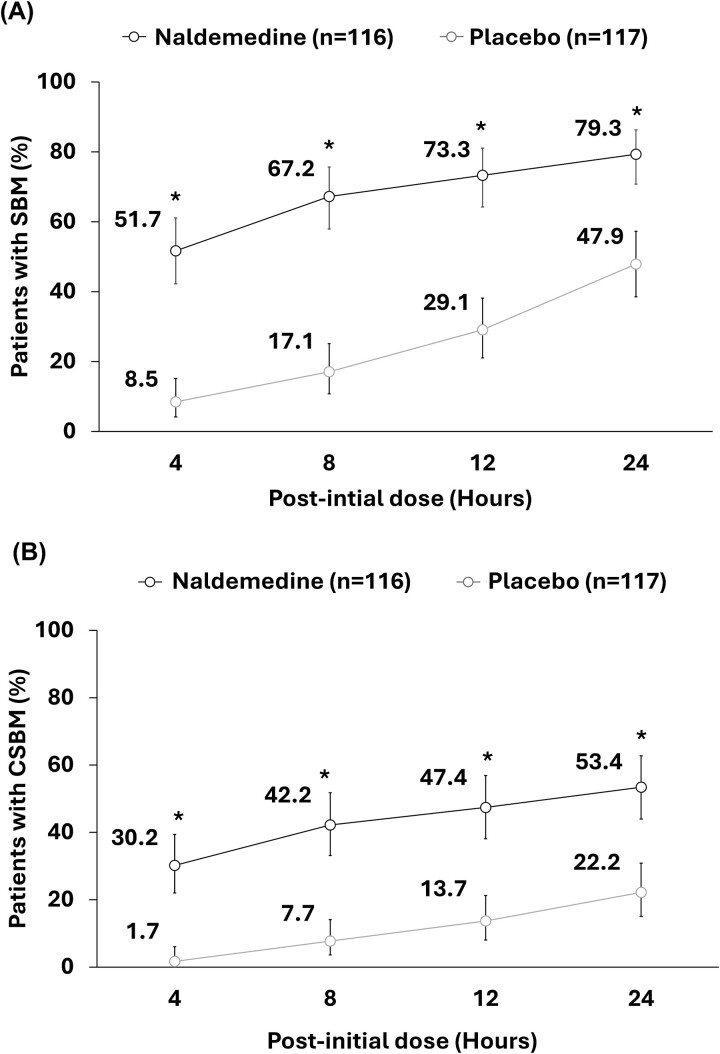
Proportion of patients with ≥1 SBM (A) or ≥1 CSBM (B) at specific time points within 24 h after initial dose of naldemedine (proportion ± 95% CI). ^*^P < 0.0001 vs. placebo. CI, confidence interval; CSBM, complete spontaneous bowel movement; SBM, spontaneous bowel movement.

Of 116 patients in the naldemedine group and 117 patients in the placebo group, 51.7% (60/116) vs. 8.5% (10/117) patients reported ≥1 SBM at 4 h after initial dose of the respective intervention. Correspondingly, 79.3% (92/116) vs. 47.9% (56/117) patients reported ≥1 SBM at 24 h (Fig. 3). In the naldemedine vs. placebo group, 30.2% (35/116) vs. 1.7% (2/117) patients reported ≥1 CSBM at 4 h after the initial dose of respective intervention (Fig. 3). Corresponding proportions for ≥1 CSBM at 24 h after the initial dose were 53.4% (62/116) vs. 22.2% (26/117), respectively (Fig. 3). Significant differences (*P* < 0.0001) in proportions of patients with ≥1 SBM and ≥1 CSBM were observed between the naldemedine group and placebo group at all time points (4, 8, 12 and 24 h).

**Table 3:** Changes of frequencies of SBMs, CSBM, SBMs with Bristol stool scale of 3 or 4 and without straining

		**Naldemedine group** **(*n* = 116)**		**Placebo group** **(*n* = 117)**		***P* value**
		**Mean/** **LS mean**	**SD/SE**	**Mean/** **LS mean**	**SD/SE**	
SBMs (per week)	Baseline	0.97	0.81	1.12	0.83	
	Treatment period	6.10	6.59	2.93	2.73	
	Change from baseline	5.09	0.47	1.79	0.47	<0.0001
CSBMs (per week)	Baseline	0.44	0.62	0.45	0.64	
	Treatment period	3.52	3.80	1.22	1.72	
	Change from baseline	3.08	0.27	0.77	0.27	<0.0001
SBMs without straining (per week)	Baseline	0.44	0.60	0.45	0.62	
	Treatment period	4.28	6.59	1.56	2.07	
	Change from baseline	3.80	0.45	1.03	0.45	<0.0001
SBMs with Bristol stool scale score 3 or 4 (per week)	Baseline	0.39	0.58	0.39	0.59	
	Treatment period	1.99	2.44	1.31	1.85	
	Change from baseline	1.61	0.19	0.95	0.19	0.0137

Data for baseline and treatment period are expressed as mean with SD. Data for change from baseline are expressed as LS mean with SE.

The ANCOVA model has terms for treatment group as a fixed effect and baseline value as a covariate. In pooled studies, STUDY is added as a covariate factor.

SBM, spontaneous bowel movement; CSBM, complete spontaneous bowel movement; LS mean, least square mean; SD, standard deviation; SE, standard error; ANCOVA, analysis of covariance.

**Table 4:** PAC-SYM and PAC-QOL scores

**Item**			**Naldemedine group** **(*n* = 63)**		**Placebo group** **(*n* = 67)**		***P* value**
			**Mean**	**SD/95% CI**	**Mean**	**SD/95% CI**	
PAC-SYM score	Baseline	Abdominal	0.99	0.70	1.12	0.62	-
		Rectal	0.66	0.77	0.68	0.69	-
		Stool	1.35	0.83	1.52	0.91	-
		Overall	1.06	0.63	1.18	0.64	-
	Change from baseline	Abdominal	−0.13	0.65	−0.19	0.45	0.5758
		Rectal	−0.21	0.81	−0.21	0.61	0.9819
		Stool	−0.48	0.81	−0.26	0.47	0.1185
		Overall	−0.3	−0.91	−0.22	−0.66	0.4719
PAC-SYM responder % (n/n)			10.9 (6/55)	4.11, 22.25	3.4 (2/59)	0.41, 11.71	0.1163
PAC-QOL score	Baseline	Physical discomfort	1.10	0.70	1.16	0.65	-
		Psychological discomfort	0.49	0.47	0.65	0.62	-
		Worries and concerns	1.11	0.67	1.14	0.71	-
		Dissatisfaction	2.56	0.80	2.61	0.69	-
		Overall	1.19	0.51	1.26	0.56	-
	Change from baseline	Physical discomfort	−0.47	0.74	−0.17	0.64	0.0216
		Psychological discomfort	−0.17	0.41	−0.08	0.46	0.2847
		Worries and concerns	−0.28	0.66	−0.12	−0.52	0.1493
		Dissatisfaction	−0.56	0.98	−0.16	0.90	0.0251
		Overall	−0.33	0.52	−0.12	0.43	0.0255
PAC-QOL responder % (n/n)			38.2% (21/55)	25.41%, 52.27%	16.9% (10/59)	8.44%, 28.97%	0.0109

Data for PAC-SYM and PAC-QOL scores are expressed as mean and SD.

Data for PAC-SYM responder and PAC-QOL responder are expressed as responder rate (%) with number of patients (responder/total) and 95% CI.

PAC-SYM responders are those with improved PAC-SYM overall score ≥1 from baseline.

PAC-QOL responders are those with improved PAC-QOL dissatisfaction score ≥1 from baseline.

95% CI is calculated by using Clopper–Pearson method.

*P* values for changes from PAC-SYM baseline score and changes from PAC-QOL baseline score are from Welch t-test, *P* values for PAC-SYM responder and PAC-QOL responder are from the chi-square test.

PAC-SYM, patients’ assessment of constipation symptoms; PAC-QOL, patients’ assessment of constipation-quality of life; SD, standard deviation; CI, confidence interval.

**Table 5:** Summary of adverse events in safety evaluation

**Type of event**	**Naldemedine group** **(*n* = 116)**	**Placebo group** **(*n* = 117)**	***P* value** *
Adverse events	63 (54.3)	44 (37.6)	0.0126
Deaths	1 (0.9)	0 (0.0)	0.4979
Serious adverse events except deaths	4 (3.4)	2 (1.7)	0.4460
Significant adverse events	8 (6.9)	8 (6.8)	1.0000
Adverse events leading to withdrawal	5 (4.3)	1 (0.9)	0.1194
Diarrhoea	34 (29.3)	20 (17.1)	0.0303
Severe diarrhoea	2 (1.7)	0 (0.0)	0.2468

Data are expressed as *n* (%).

^*^Significance was calculated using Fisher exact test between naldemedine and placebo groups.

### Changes in frequencies of SBMs, CSBM, SBMs with BSS of 3 or 4 and without straining

Changes in frequency of SBMs per week from baseline (LS mean ± SE) were 5.09 ± 0.47 in the naldemedine group vs. 1.79 ± 0.47 for the placebo group (*P* < 0.0001). Similarly, changes in frequency of CSBMs per week from baseline (LS mean ± SE) were 3.08 ± 0.27 in the naldemedine group vs. 0.77 ± 0.27 for the placebo group (*P* < 0.0001) (Table 3). Changes in frequencies of SBMs without straining per week from baseline (LS mean ± SE) were 3.80 ± 0.45 for the naldemedine group, which was significantly higher (*P* < 0.0001) than for the placebo group (1.03 ± 0.45). Changes in frequencies of SBMs with BSS 3 and 4 per week (LS mean ± SE) from baseline were 1.61 ± 0.19 for the naldemedine group, which was significantly higher than 0.95 ± 0.19 for the placebo group (*P* < 0.05; Table 3). Changes in number of days with at least 1 SBM or 1 CSBM per week from baseline during the 2-week treatment period (LS mean ± SE) were significantly higher in the naldemedine group (2.79 ± 0.16 and 1.98 ± 0.15, respectively) vs. placebo group (1.15 ± 0.16 and 0.59 ± 0.15, respectively) (both *P* < 0.0001; Table S3).

### PAC-SYM score

At baseline, overall PAC-SYM score (mean ± SD) was 1.06 ± 0.63 for the naldemedine group and 1.18 ± 0.64 for the placebo group (Table 4); the corresponding changes from baseline (−0.3 ± 0.91 vs. −0.22 ± 0.66) were not significant (*P* = 0.4719). In addition, changes from baseline in individual domain scores for PAC-SYM were comparable between groups. PAC-SYM responder rates (10.9 vs. 3.4%) were not significantly different (*P* = 0.1163) in naldemedine vs. placebo groups.

### PAC-QOL score

At baseline, overall PAC-QOL score (mean ± SD) was 1.19 ± 0.51 for the naldemedine group and 1.26 ± 0.56 for the placebo group (Table 4). Overall change in PAC-QOL baseline score (mean ± SD) was −0.33 ± 0.52 for the naldemedine group, which was significantly larger (*P* < 0.05) than for the placebo group (−0.12 ± 0.43) (Table 4). Similarly, PAC-QOL responder rate was significantly higher (*P* < 0.05) in naldemedine (38.2%) vs. placebo (16.9%) groups. PAC-QOL dissatisfaction domain score also significantly improved from baseline (*P* < 0.05) for naldemedine (−0.56 ± 0.98) vs. placebo (−0.16 ± 0.90) groups (Table 4).

### Safety

Of 116 patients in the naldemedine group, 63 (54.3%) patients reported AEs, 1 (0.9%) patient died, 4 (3.4%) patients reported SAEs except deaths, 8 (6.9%) patients experienced significant AEs and 5 (4.3%) patients reported AEs leading to withdrawal of naldemedine treatment. Furthermore, 34 (29.3%) patients reported diarrhoea and 2 (1.7%) patients reported severe diarrhoea (Table 5). Of 117 patients in placebo group, 44 (37.6%) patients reported AEs, 2 (1.7%) patients reported SAEs except death, 8 (6.8%) patients experienced significant AEs, 1 (0.9%) patient reported AEs leading to withdrawal of placebo treatment, 20 (17.1%) patients reported diarrhoea and no patients reported severe diarrhoea (Table 5). Incidences of AEs and diarrhoea were significantly higher (*P* < 0.05) in the naldemedine group compared with the placebo group. Incidences of death, SAEs, significant AEs, AEs which led to treatment withdrawal and severe diarrhoea were comparatively higher in the naldemedine group than in the placebo group; however, the differences were not statistically significant.

## Discussion

This pooled *post hoc* subgroup analysis was conducted using the data from patients who participated in the previous phase IIb and III clinical trials of naldemedine targeting cancer patients with OIC, and had been taking magnesium oxide before starting naldemedine and continued its use during naldemedine treatment. The results of this study showed significant improvement (*P* < 0.0001) in the frequency of SBMs, CSBMs, SBM responder rate and the CSBM responder rate in patients in the naldemedine group compared with those in the placebo group. This study also showed significant improvement (*P* < 0.05) in PAC-QOL scores in patients in the naldemedine group compared with those who received only placebo. These results are generally consistent with the results observed in the phase IIb and III studies. Furthermore, the results suggested that the effect of naldemedine was independent of magnesium oxide as its mechanism of action is different. Magnesium oxide does not have a direct effect on the cause of the OIC, while naldemedine, being a PAMORA, acts through inhibiting peripherally acting MOR. Moreover, frequencies of AEs were similar to previously conducted phase III and pooled studies (23,25). This suggested that naldemedine was tolerable and had an acceptable safety profile and no additional concerns were raised with add-on naldemedine therapy in cancer patients who previously used magnesium oxide for the treatment of OIC as a first-line medication.

OIC is the most common side effect associated with the use of opioid in patients who are on cancer treatment (18). In addition, OIC impacts the well-being and QOL of cancer patients with OIC (7). Constipation can lead to serious complications if it is underestimated or untreated. Therefore, it is extremely important to manage constipation in clinical practice using early therapeutic interventions (12). There are two possible treatment strategies for OIC: initial treatment with non-specific laxatives, such as magnesium oxide, followed by specific medication, such as naldemedine, as it is generally followed in actual clinical practice in Japan (15) or initial treatment with specific medication, such as naldemedine, followed by non-specific laxatives, such as magnesium oxide, as is suggested by a previous retrospective database study (27).

A single-centre, open-label, two-arm, phase II randomized controlled trial has compared incidence of OIC when opioids were administered concomitantly with magnesium oxide vs. naldemedine for 2 weeks, and showed that the number of patients diagnosed with constipation by the Rome IV criteria was significantly lower (*P* < 0.001) in the naldemedine group than in the magnesium oxide group (28). They also demonstrated that the naldemedine group had lesser deterioration of PAC-QOL, PAC-SYM and CSBMs, and lower incidences of nausea compared with the magnesium oxide group (28). Since the actual cause of OIC is clear and its incidence is high in patients taking opioids for pain management, it is reasonable to administer a PAMORA simultaneously with opioids (12). Conversely, magnesium oxide is the most common and affordable osmotic laxative drug in Japan and many patients prefer to use it for the treatment of acute or chronic constipation even before starting opioid treatment (13,18). Therefore, it is important to consider treatment strategies for patients who had been taking magnesium oxide initially but had not shown substantial improvement in OIC symptoms. It may be speculated that magnesium oxide may have limited efficacy in OIC despite having considerable efficacy in constipation of other etiologies (16).

Our results are also consistent with the previous study that showed a significant difference in the proportions of SBM responders between the naldemedine and placebo groups (*P* < 0.0001) in pooled analysis (25). This demonstrated the effectiveness of naldemedine for OIC treatment, independent of prior and continued magnesium oxide use, in cancer patients. Since magnesium oxide is a non-selective laxative, it must have led to improvement and control over constipation other than OIC. Thus, it might be possible that effectiveness of add-on naldemedine was more pronounced in these patients as other constipation might have been treated with prior and ongoing magnesium oxide treatment. This is further supported by the results of a previous post-marketing surveillance study which reported the greatest improvement in the frequency and condition of bowel movements in the naldemedine group with prior and concomitant use of osmotic or saline laxatives (28). These results support the speculation that prior and concomitant treatment with other laxatives manages constipation unrelated to opioid use, while naldemedine effectively manages the remaining OIC.

In the previous phase III trial, only the dissatisfaction domain score of PAC-QOL was significantly improved (*P* = 0.015) in the naldemedine group compared with the placebo group (23), while this study showed significant improvement (*P* < 0.05) in the overall PAC-QOL score, physical discomfort domain score, dissatisfaction domain score and the PAC-QOL responder rate in the naldemedine group compared with the placebo group. This indicates that the improvement of OIC symptoms would provide the resultant improvement in the patient-reported outcomes of QOL. The results of this study demonstrated that overall mean PAC-SYM score from baseline was not significantly different between naldemedine and placebo groups, similar to the previous study (23). It must be noted that the PAC-SYM baseline scores were relatively low (mean score ~1.00), possibly resulting in smaller effect sizes that were not significantly different between the treatment groups.

Although AEs and diarrhoea were significantly higher (*P* < 0.05) in naldemedine vs. placebo groups, no significant differences were observed in the incidences of SAEs, death, AEs leading to treatment withdrawal and diarrhoea between the two groups. This suggested that no additional safety concerns by naldemedine treatment added to the ongoing magnesium oxide treatment. Furthermore, previous studies also reported that the incidence of diarrhoea was not significantly affected by prior and concomitant laxative use (29,30). Similarly, results of this study reported that naldemedine use, concomitantly with magnesium oxide, did not affect the incidences of SAEs and severe diarrhoea compared with the placebo group receiving magnesium oxide.

This study demonstrated that administering add-on naldemedine to the ongoing magnesium oxide treatment in cancer patients with OIC was efficacious and tolerable, similar to the phase III study (23). The recent database study (27), which adjusted baseline characteristics by propensity score matching, reported that addition, change or dose increase of laxatives was comparable between the naldemedine and magnesium oxide groups during initial treatment. However, the study reported a trend to prescribe naldemedine with more severe conditions, suggesting that baseline matching was not sufficient. Further research is needed to determine the possible treatment algorithms of the first-line treatment with naldemedine or magnesium oxide, probably considering patient and disease characteristics.

## Strengths and limitations

This is the first study exploring the effect and safety of naldemedine in cancer patients with prior and concomitant use of magnesium oxide for OIC treatment, in Japan. However, in this pooled *post hoc* subgroup analysis, patients were not randomly allocated, especially in terms of dosage of magnesium oxide and duration of prior use. Moreover, this study included a relatively smaller patient population compared with the previously published clinical trials conducted in Japan and Korea. Hence, the results may have limited generalizability to diverse global populations.

## Conclusion

This is the first study that demonstrated the add-on effect of naldemedine as a concomitant treatment in Japanese patients with cancer and insufficient response to prior magnesium oxide therapy for the treatment of OIC. This pooled, subgroup analysis also demonstrated the safety profile of naldemedine in cancer patients receiving prior magnesium oxide as a first-line medication for the treatment of OIC.

## Supplementary Material

Figure_S1_hyae135

6TABLE_1__hyae135

Table_S2_hyae135

Table_S3_hyae135

## Data Availability

The data cannot be made publicly available due to restrictions as sharing of data could compromise the privacy of research participants.

## Clinical trial registration number

This study comprised data of a previously published phase IIb study (Clinical trial registration number: jRCT2080221471) and a phase III COMPOSE-4 study (jRCT2080222291).
