# Supplementary material for: Effect of add-on naldemedine treatment in patients with cancer and opioid-induced constipation insufficiently responding to magnesium oxide: a pooled, subgroup analysis of two randomized controlled trials
Source: Jpn J Clin Oncol. 2024 Oct 1;55(1):40–8. doi: 10.1093/jjco/hyae135 (PMC11708229; doi:10.1093/jjco/hyae135)
Supplement: Figure_S1_hyae135 [file figure_s1_hyae135.docx]

**Figure S1. CONSORT diagram for creation of pooled naldemedine and placebo groups**


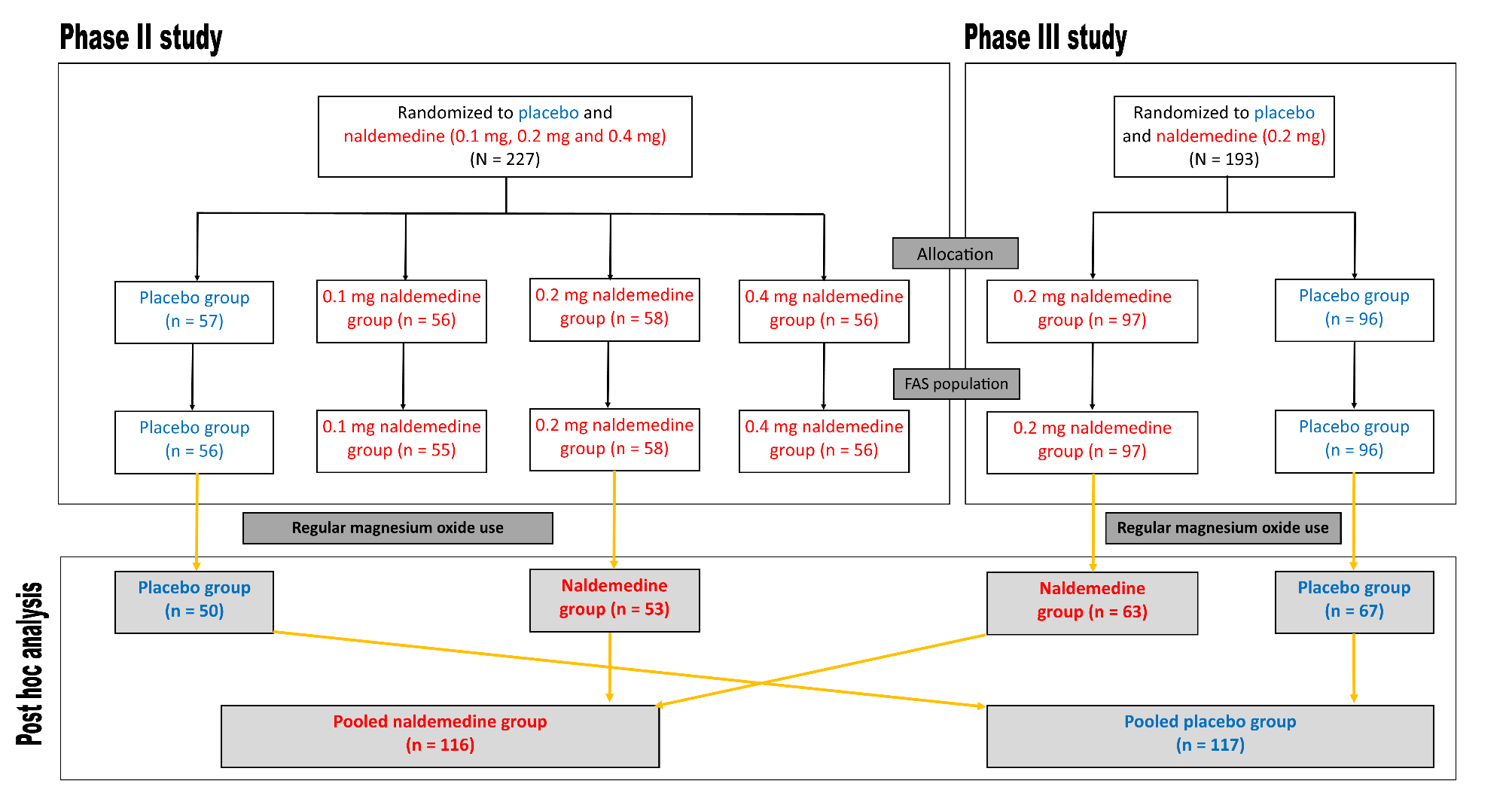


Regular magnesium oxide use: Patients who had been using magnesium oxide before starting naldemedine or placebo and continued its use concomitantly with naldemedine or placebo.
