# Supplementary material for: Effect of add-on naldemedine treatment in patients with cancer and opioid-induced constipation insufficiently responding to magnesium oxide: a pooled, subgroup analysis of two randomized controlled trials
Source: Jpn J Clin Oncol. 2024 Oct 1;55(1):40–8. doi: 10.1093/jjco/hyae135 (PMC11708229; doi:10.1093/jjco/hyae135)
Supplement: 6TABLE_1__hyae135 [file 6table_1__hyae135.docx]

**Table S1. Patient demographic and baseline characteristics from previous studies.**

| **Characteristic** | **Phase IIb study** | | **Phase III study** | |
| --- | --- | --- | --- | --- |
|  | **Naldemedine group**  **(n=53)** | **Placebo group (n=50)** | **Naldemedine group (n=63)** | **Placebo group**  **(n=67)** |
| *Age, years* | | | | |
| Mean (SD) | 63.2 (10.4) | 63.9 (9.8) | 63.9 (9.5) | 65.4 (11.4) |
| *Age categories, n (%), years* | | | | |
| <40 | 3 (5.7) | 1 (2.0) | 1 (1.6) | 1 (1.5) |
| ≥40 to <50 | 3 (5.7) | 4 (8.0) | 5 (7.9) | 7 (10.4) |
| ≥50 to <65 | 21 (39.6) | 18 (36.0) | 25 (39.7) | 21 (31.3) |
| ≥65 to <75 | 23 (43.4) | 23 (46.0) | 25 (39.7) | 18 (26.9) |
| ≥75 | 3 (5.7) | 4 (8.0) | 7 (11.1) | 20 (29.9) |
| *Sex, n (%)* | | | | |
| Male | 32 (60.4) | 30 (60.0) | 38 (60.3) | 44 (65.7) |
| Female | 21 (39.6) | 20 (40.0) | 25 (39.7) | 23 (34.3) |
| *Body weight (kg)* | | | | |
| Mean (SD) | 56.7 (7.6) | 56.4 (11.0) | 53.9 (10.5) | 54.1 (11.0) |
| *Body mass index (kg/m^2^)* | | | | |
| Mean (SD) | 22.0 (3.0) | 21.6 (3.8) | 21.2 (3.7) | 20.7 (3.7) |
| *Body mass index categories, n (%)* | | | | |
| <18.5 | 8 (15.1) | 8 (16.0) | 12 (19.0) | 20 (29.9) |
| ≥18.5 to <25 | 35 (66.0) | 35 (70.0) | 43 (68.3) | 38 (56.7) |
| ≥25 to <30 | 10 (18.9) | 5 (10.0) | 6 (9.5) | 8 (11.9) |
| ≥30 | 0 | 2 (4.0) | 2 (3.2) | 1 (1.5) |
| *Race, n (%)* | | | | |
| Asian | 53 (100.0) | 50 (100.0) | 63 (100.0) | 67 (100.0) |
| *Regular opioid use per day at baseline*^†^*, mg (%)* | | | | |
| Mean (SD) | 81.6 (87.8) | 88.2 (102.7) | 62.2 (50.0) | 75.1 (110.3) |
| *Regular opioid use per day at baseline categories, n (%)* | | | | |
| <30 | 14 (26.4) | 10 (20.0) | 15 (23.8) | 16 (23.9) |
| ≥30 to <60 | 15 (28.3) | 15 (30.0) | 15 (23.8) | 24 (35.8) |
| ≥60 to <120 | 11 (20.8) | 13 (26.0) | 24 (38.1) | 17 (25.4) |
| ≥120 | 13 (24.5) | 12 (24.0) | 9 (14.3) | 10 (14.9) |
| *Use of rescue laxative per week at baseline (times)* | | | | |
| Mean (SD) | 6.3 (4.9) | 7.0 (5.3) | 5.3 (3.7) | 5.3 (4.5) |
| *Country, n (%)* | | | | |
| Japan | 50 (94.3) | 49 (98.0) | 63 (100.0) | 67 (100.0) |
| Korea | 3 (5.7) | 1 (2.0) | 0 | 0 |
| *Category of patient, n (%)* | | | | |
| Inpatient | 14 (26.4) | 14 (28.0) | 13 (20.6) | 15 (22.4) |
| Outpatient | 39 (73.6) | 36 (72.0) | 50 (79.4) | 52 (77.6) |
| *Primary tumor diagnosed, n (%)* | | | | |
| Lung | 17 (32.1) | 28 (56.0) | 26 (41.3) | 31 (46.3) |
| Breast | 12 (22.6) | 13 (26.0) | 13 (20.6) | 13 (19.4) |
| Large intestine | 3 (5.7) | 0 | 1 (1.6) | 1 (1.5) |
| Other | 21 (39.6) | 9 (18.0) | 23 (36.5) | 22 (32.8) |
| *Presence of metastasis, n (%)* | | | | |
| Yes | 48 (90.6) | 46 (92.0) | 51 (81.0) | 60 (89.6) |
| No | 5 (9.4) | 4 (8.0) | 12 (19.0) | 7 (10.4) |
| *Eastern Cooperative Oncology Group performance status, n (%)* | | | | |
| 0 | 11 (20.8) | 15 (30.0) | 14 (22.2) | 24 (35.8) |
| 1 | 30 (56.6) | 30 (60.0) | 39 (61.9) | 32 (47.8) |
| 2 | 12 (22.6) | 5 (10.0) | 10 (15.9) | 11 (16.4) |

^†^Dose of opioid analgesics was used by converting into equivalent oral morphine.

SD, standard deviation.
