# Supplementary material for: Effect of add-on naldemedine treatment in patients with cancer and opioid-induced constipation insufficiently responding to magnesium oxide: a pooled, subgroup analysis of two randomized controlled trials
Source: Jpn J Clin Oncol. 2024 Oct 1;55(1):40–8. doi: 10.1093/jjco/hyae135 (PMC11708229; doi:10.1093/jjco/hyae135)
Supplement: Table_S2_hyae135 [file table_s2_hyae135.docx]

**Table S2. Regular and rescue laxatives used in the naldemedine and placebo group.**

| **Type of laxatives** | **Naldemedine group**  **(n=116)** | **Placebo group (n=117)** |
| --- | --- | --- |
| *Regular laxatives,* n (%)* | | |
| Magnesium oxide | 116 (100) | 117 (100) |
| Sennosides | 12 (10.3) | 17 (14.5) |
| Pantethine | 9 (7.8) | 10 (8.5) |
| Sodium picosulfate | 2 (1.7) | 2 (1.7) |
| Senna leaf/pods granules | 1 (0.9) | 2 (1.7) |
| Lubiprostone | - | 2 (1.7) |
| Lactose | 1 (0.9) | - |
| Mashiningan | 1 (0.9) | - |
| Polycarbophil | 1 (0.9) | - |
| Daikenchuto^†^ | - | 1 (0.9) |
| Mosapride | - | 1 (0.9) |
| Tokakujokito^ | - | 1 (0.9) |
| *Rescue laxatives,** *n (%)* |  |  |
| Sennosides | 29 (25.0) | 44 (37.6) |
| Magnesium oxide | 22 (19.0) | 34 (29.1) |
| Sodium picosulfate | 17 (14.7) | 20 (17.1) |
| Glycerin | 3 (2.6) | 6 (5.1) |
| New lecicarbon | 2 (1.7) | 7 (6.0) |
| Pantethine | 3 (2.6) | 3 (2.6) |
| Lactulose | 2 (1.7) | 2 (1.7) |
| Glycerol | 1 (0.9) | 2 (1.7) |
| Laxatives | 1 (0.9) | 2 (1.7) |
| Bisacodyl | 1 (0.9) | 2 (1.7) |
| Lubiprostone | 1 (0.9) | - |
| Sodium bicarbonate | 1 (0.9) | - |
| Calcium sennoside+ Bisacodyl | 1 (0.9) | - |
| Senna leaf/pods granules | - | 3 (2.6) |
| Contact laxatives (Enema) | - | 1 (0.9) |
| Senna supplements | - | 1 (0.9) |

*Duplicate

^†^Daikenchuto is made from a mixture of herbal extract powder and maltose, and contains processed ginger, ginseng, and Japanese pepper.

^Tokakujokito is an extract made from peach kernel, cinnamon bark, rhubarb, glycyrrhiza, and mirabilitum.
