# Supplementary material for: Effect of add-on naldemedine treatment in patients with cancer and opioid-induced constipation insufficiently responding to magnesium oxide: a pooled, subgroup analysis of two randomized controlled trials
Source: Jpn J Clin Oncol. 2024 Oct 1;55(1):40–8. doi: 10.1093/jjco/hyae135 (PMC11708229; doi:10.1093/jjco/hyae135)
Supplement: Table_S3_hyae135 [file table_s3_hyae135.docx]

**Table S3. Analysis of change in number of days with at least 1 SBM per week from baseline during 2-week treatment period.**

|  | | **Naldemedine group**  **(n=116)** | | **Placebo group (n=117)** | | ***P*-value** |
| --- | --- | --- | --- | --- | --- | --- |
|  |  | **Mean/ LS mean** | **SD/SE** | **Mean/ LS mean** | **SD/SE** |  |
| SBMs  (days/week) | Baseline | 0.87 | 0.74 | 1.02 | 0.76 |  |
|  | Treatment period | 3.66 | 2.04 | 2.16 | 1.71 |  |
|  | Change from baseline | 2.79 | 0.16 | 1.15 | 0.16 | <0.0001 |
| CSBMs  (days/week) | Baseline | 0.41 | 0.56 | 0.41 | 0.57 |  |
|  | Treatment period | 2.39 | 2.03 | 1.00 | 1.31 |  |
|  | Change from baseline | 1.98 | 0.15 | 0.59 | 0.15 | <0.0001 |

Data for baseline and treatment period are expressed as mean with SD. Data for change from baseline are expressed as LS mean with SE.

The ANCOVA model has terms for treatment group as a fixed effect and baseline value as a covariate. In pooled studies, STUDY is added as a covariate factor.

ANCOVA, analysis of covariance; CSBM, complete spontaneous bowel movement; LS mean, least square mean; SBM, spontaneous bowel movement; SD, standard deviation; SE, standard error
